# Increased Expression of EZH2 Is Mediated by Higher Glycolysis and mTORC1 Activation in Lupus CD4^+^ T Cells

**DOI:** 10.20900/immunometab20200013

**Published:** 2020-04-09

**Authors:** Xiaoqing Zheng, Pei-Suen Tsou, Amr H. Sawalha

**Affiliations:** 1 Division of Rheumatology, Department of Pediatrics, Children’s Hospital of Pittsburgh, University of Pittsburgh, Pittsburgh, PA 15260, USA; 2 Division of Rheumatology, Department of Internal Medicine, University of Michigan, Ann Arbor, MI 48109, USA; 3 Division of Rheumatology and Clinical Immunology, Department of Medicine, University of Pittsburgh, Pittsburgh, PA 15260, USA; 4 Lupus Center of Excellence, University of Pittsburgh School of Medicine, Pittsburgh, PA 15213, USA

**Keywords:** EZH2, epigenetics, lupus, CD4^+^ T cells, glycolysis, immunometabolism, autoimmunity

## Abstract

**Objective:**

EZH2 is overexpressed in CD4^+^ T cells from patients with systemic lupus erythematosus (SLE). Increased disease activity in SLE patients is associated with a proinflammatory epigenetic shift in naïve CD4^+^ T cells, likely mediated by EZH2. Here we aim to understand the upstream mechanisms underlying EZH2 overexpression in SLE CD4^+^ T cells.

**Methods:**

Naïve CD4^+^ T cells were isolated from SLE patients and then stimulated with anti-CD3/anti-CD28. qPCR and Western blotting were used to measure mRNA and protein expression levels, respectively. 2-Deoxy-d-glucose (2-DG) was used to inhibit glycolysis. mTORC1 signaling was inhibited using rapamycin. Oxidative stress was induced by H_2_O_2_.

**Results:**

Because glycolysis is increased in SLE CD4^+^ T cells and glycolysis regulates miR-26a and miR-101, which target EZH2, we examined the effect of inhibiting glycolysis on EZH2 expression. 2-DG significantly inhibited EZH2 expression in SLE CD4^+^ T cells. In addition, 2-DG restored the expression of miR-26a and miR-101, suggesting that suppression of EZH2 by 2-DG occurs at the post-transcriptional level. Because mTORC1 is activated in SLE CD4^+^ T cells in part due to increased oxidative stress, and mTORC1 activation increases glycolysis, we hypothesized that mTORC1 mediates increased EZH2 expression. Indeed, inhibiting mTORC1 increased miR-26a and miR-101 and suppressed EZH2 expression in SLE CD4^+^ T cells. Further, H_2_O_2_ treatment increased EZH2 expression, however, this effect appears to be independent of miR-26a and miR-101.

**Conclusion:**

Increased EZH2 is mediated by activation of mTORC1 and increased glycolysis in SLE CD4^+^ T cells. Therapeutic effects from inhibiting mTOR or glycolysis in SLE might be in part mediated by suppression of EZH2.

## INTRODUCTION

Systemic lupus erythematosus (SLE) is a chronic remitting-relapsing autoimmune disease characterized by T cell autoreactivity and autoantibody production. The pathogenesis of SLE is incompletely understood, however, genetic, epigenetic, and environmental factors contribute to SLE development [1]. A substantial body of evidence supports a key role for epigenetic dysregulation in the pathogenesis of SLE [2].

We have previously demonstrated that proinflammatory epigenetic changes occur in naïve CD4^+^ T cells as the disease becomes more active in SLE patients. These epigenetic changes precede corresponding transcriptional changes and poise naïve CD4^+^ T cells for Th2/Th17/Tfh immune responses, while suppressing regulatory T cell pathways such as TGF-β signaling [3]. We also provided evidence to suggest that this “epigenetic shift” is likely mediated by the enhancer of zeste homolog 2 (EZH2), which is a core component of polycomb repressive complex 2 (PRC2) and mediates tri-methylation of lysine 27 on histone 3 (H3K27me3) [3]. Indeed, we subsequently demonstrated that CD4^+^ T cells from SLE patients are characterized by increased expression of EZH2 and higher levels of H3K27me3 compared to healthy controls [4]. In addition, levels of two microRNAs, miR-26a, and miR-101, which are known to target and downregulate EZH2 were reduced in SLE CD4^+^ T cells [4]. Further, inhibiting EZH2 significantly improved survival and ameliorated lupus-like disease in MRL/*lpr* lupus-prone mice [5].

The mechanisms underlying EZH2 upregulation in SLE CD4^+^ T cells remain unknown. It has been demonstrated that in cancer-infiltrating T cells the expression of miR-26a and miR-101 is sensitive to glucose availability and glycolysis [6]. Recent evidence suggests that glycolysis is increased in SLE T cells and that restoring normal glucose metabolism might be of therapeutic benefit [7]. In addition, the mechanistic target of rapamycin complex 1 (mTORC1) has been shown to be a metabolism sensor in immune cells [8], and is activated in CD4^+^ T cells from SLE patients [9]. Blocking mTOR activation was associated with promising clinical and cellular response in SLE patients [10]. In this study, we explore the relationship between glycolysis, mTORC1 signaling, and EZH2 expression in SLE CD4^+^ T cells.

## METHODS

### SLE Patients

SLE patients were recruited from the Lupus Center of Excellence at the University of Pittsburgh Medical Center and from the University of Michigan rheumatology clinics. All patients included in this study met the American College of Rheumatology classification criteria for SLE [11]. Systemic lupus erythematosus disease activity index (SLEDAI) scores of the patients ranged from 0 to 12, with a mean of 3.3 and a median of 2. Demographic information for SLE patients included in this study are shown in Supplementary Table S1. All subjects included in this study signed a written informed consent approved by the Institutional Review Board of the University of Pittsburgh (STUDY19020379; date approved: 5/16/2019) and the University of Michigan (HUM00061490; date approved: 5/15/2012).

### Naïve CD4^+^ T Cells Isolation and Culture

Naïve CD4^+^ T cells were isolated from fresh human blood samples with a negative selection isolation kit from Miltenyi Biotec as per protocol. The purity of naïve CD4^+^ T cells was evaluated with staining of anti-CD3 (clone UCHT1, BioLegend, San Diego, USA), anti-CD4 (clone RPA-T4, BioLegend, San Diego, USA), and anti-CD45RA (HI100, BioLegend, San Diego, USA). Isolated cell purities were over 95% (Supplementary Figure S1). Cells were cultured in RPMI 1640 media (GE Health Care Life Sciences, Marlborough, USA) supplemented with 10% FBS (Life Technologies, Carlsbad, USA). Cells were stimulated overnight with anti-CD3 (10 μg/mL, pre-coated on plate, Clone UCHT1, BD Biosciences, San Jose, USA) and anti-CD28 (2.5 μg/mL, Clone CD28.2, BD Biosciences, San Jose, USA), with and without glycolysis inhibitor 2-deoxy-d-glucose (2-DG, 2 mg/mL, Acros Organics, New Jersey, USA), mTOR inhibitor rapamycin (100 Nm, Alfa Aesar, Ward Hill, USA) or H_2_O_2_ (50 μM, Sigma-Aldrich, St. Louis, USA). The next day the antibodies were removed and fresh media (RPMI and 10% FBS) were added with and without 2-DG, rapamycin, or H_2_O_2_. The cells were cultured for a total of 3 days before harvesting.

### RNA Isolation and Real-Time PCR Analysis

Total RNA was isolated with Direct-zol RNA MiniPrep kit (Zymo Research, Irvine, USA). cDNA was synthesized with Verso cDNA synthesis kit (Thermo Fisher Scientific, Waltham, USA) following the manufacturer’s instructions. EZH2, mTOR, and beta-actin primers were predesigned by Sigma-Aldrich (St. Louis, USA). RPL13A primers were purchased from Integrated DNA Technologies, Inc (Coralville, USA) [12].

miRNA was measured with TaqMan advanced miRNA cDNA synthesis kit (Thermo Fisher Scientific, Waltham, USA). miR-26a (assay ID: 478788) and miR-101 (assay ID: 478620) were purchased from Life Technologies (Carlsbad, USA).

### Western Blotting

Cells were harvested and lysed in RIPA buffer with protease inhibitor cocktail (Thermo Fisher Scientific, Waltham, USA) and phosphatase inhibitor cocktail (Life Technologies, Carlsbad, USA). Protein concentrations were measured with the bicinchoninic acid (BCA) method (Thermo Fisher Scientific, Waltham, USA). Proteins were separated with 4–15% precast polyacrylamide gel (Bio-Rad Laboratories, Hercules, USA) before transferring to nitrocellulose membranes. Membranes were incubated with the following antibodies at 4 °C overnight: anti-EZH2 (Novus Biologicals (Centennial, USA), 1:100), anti-p-S6 ribosomal protein (Ser235/236, Cell Signaling Technology (Beverly, USA), 1:1000), anti-H3K27me3 (Cell Signaling Technology (Beverly, USA), 1:1000), anti-H3 (Cell Signaling Technology (Beverly, USA), 1:1000), and anti-β-actin (Cell Signaling Technology (Beverly, USA), 1:1000). Goat anti-Rabbit or anti-Mouse Alexa Fluor 680 secondary antibody (Thermo Fisher Scientific (Waltham, USA), 1:1000) was incubated with membranes for 1 h at room temperature. Images were taken with Odyssey Imaging Systems (LI-COR Biotechnology, Lincoln, USA).

### Statistics

Data were expressed as mean ± standard deviation. Differences between two groups were analyzed by Wilcoxon matched-pairs signed rank test in GraphPad Prism (version 8.2, GraphPad Software). *P* values < 0.05 were considered statistically significant.

## RESULTS

**Figure 1.**
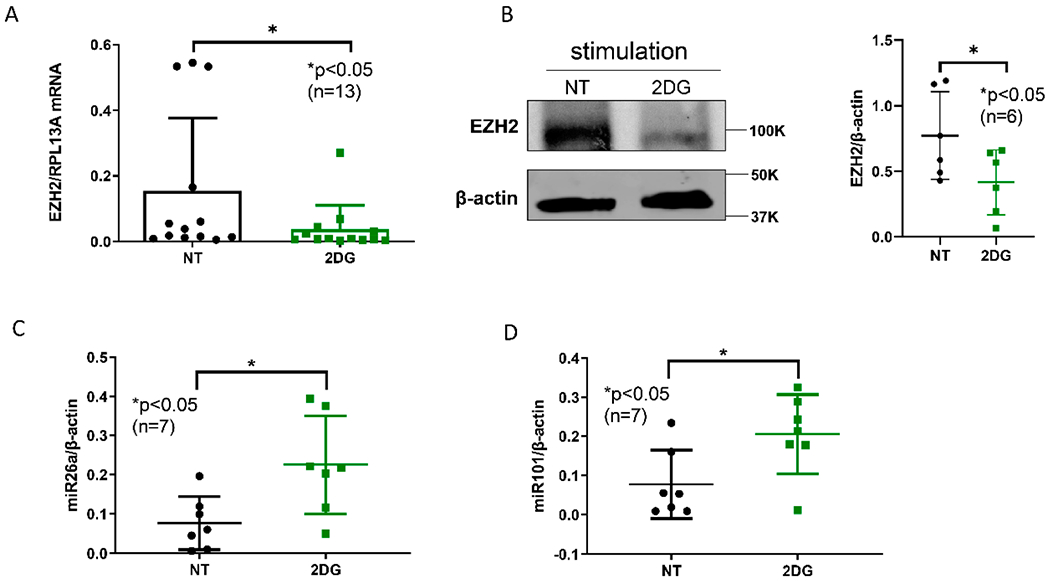
EZH2 levels were significantly reduced by glycolysis inhibitor 2-deoxy-d-glucose (2-DG) in SLE CD4^+^ T cells. (**A**) EZH2 mRNA levels were significantly decreased after 2-DG treatment in naïve CD4^+^ T cells from SLE patients. (**B**) EZH2 protein levels were significantly lower in 2-DG treated cells compared to the non-treated (NT) cells. A representative Western blot is shown from six independent samples, with quantification presented in the panel on the right. (**C**) miR-26a and (**D**) miR-101 levels were significantly higher in 2-DG treated cells compared to non-treated (NT) cells. Naïve CD4^+^ T cells were stimulated with anti-CD3 (10 μg/mL) and anti-CD 28 (2.5 μg/mL) overnight. Cells were treated with 2-DG (2 mg/mL) for three days. Data are shown as mean ± standard deviation.

To examine the effect of inhibiting glycolysis on EZH2 expression, naïve CD4^+^ T cells isolated from SLE patients were stimulated with anti-CD3/anti-CD28 in the presence or absence of glycolysis inhibitor 2-DG. Cells treated with 2-DG showed significant reduction in the mRNA expression of EZH2 (Figure 1A). A significant reduction in EZH2 protein levels with 2-DG treatment was also noted (Figure 1B). However, we did not observe significant reduction in H3K27me3 levels with 2-DG treatment (data not shown). To determine if reduced EZH2 expression with 2-DG treatment in SLE CD4^+^ T cells might be explained by an effect of 2-DG on the expression levels of miR-26a and miR-101, which regulate EZH2 and have been previously shown to be sensitive to glucose availability, we measured miR-26a and miR-101 levels in SLE CD4^+^ T cells with and without 2-DG treatment. 2-DG was associated with significant upregulation in miR-26a and miR-101 expression levels in SLE CD4^+^ T cells (Figure 1C,D). Because we have previously shown that miR-26a and miR-101 are suppressed in SLE CD4^+^ T cells compared to normal healthy controls, these data suggest that increased glycolysis might potentially explain EZH2 overexpression in SLE CD4^+^ T cells. Indeed, our data suggest that inhibiting glycolysis can restore the expression levels of miR-26a and miR-101, which regulate EZH2 expression.

**Figure 2.**
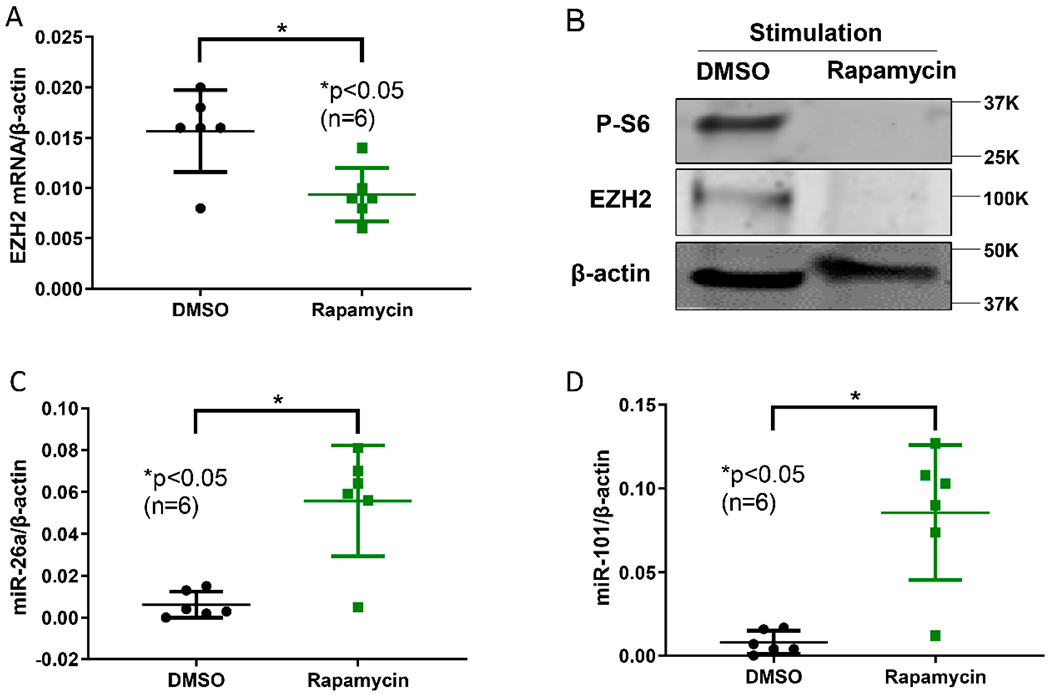
mTORC1 inhibitor rapamycin downregulated EZH2 levels in SLE CD4^+^ T cells. (**A**) EZH2 mRNA levels were significantly decreased by rapamycin. (**B**) Rapamycin treatment significantly inhibited mTORC1 as demonstrated by reduced phosphorylated S6 (p-S6), and reduced EZH2 at the protein level. A representative blot is shown from three independent samples. (**C**) miR-26a and (**D**) miR-101 levels were upregulated by rapamycin compared to DMSO (vehicle) treated cells. Naïve CD4^+^ T cells were stimulated with anti-CD3 (10 μg/mL) and anti-CD 28 (2.5 μg/mL) overnight. Cells were treated with DMSO or rapamycin (100 nM) for three days. Data are shown as mean ± standard deviation.

Since mTORC1 signaling increases glycolysis in CD4^+^ T cells [13], and mTORC1 is activated in SLE CD4^+^ T cells [14], we tested the effect of inhibiting mTORC1 on SLE CD4^+^ T cell EZH2 expression. SLE CD4^+^ T cells treated with rapamycin showed significant downregulation of EZH2 expression at the mRNA and protein levels (Figure 2A,B). In addition, rapamycin treatment resulted in upregulation of both miR-26a and miR-101 (Figure 2C,D). Taken together, these data suggest that increased mTORC1 activity in SLE CD4^+^ T cells might mediate upregulation of EZH2 through increasing glycolysis and the resulting suppression of miR-26a and miR-101.

**Figure 3.**
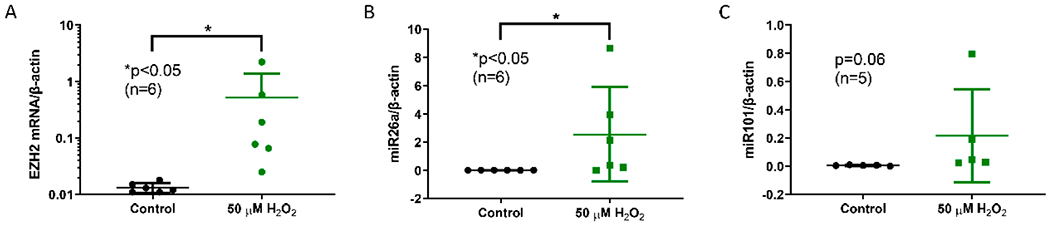
EZH2, miR-26a, and miR-101 levels with oxidative stress. (**A**) EZH2 mRNA levels were markedly increased with 50 μM H_2_O_2_ treatment. (**B**) miR-26a levels were increased by H_2_O_2_ exposure. (**C**) A trend for higher miR-101 levels with H_2_O_2_ exposure was noted. Naïve CD4^+^ T cells were stimulated with anti-CD3 (10 μg/mL) and anti-CD 28 (2.5 μg/mL) overnight. Cells were treated with and without 50 μM H_2_O_2_ for 3 days. Data are shown as mean ± standard deviation.

Oxidative stress is linked to SLE pathogenesis and disease flares in SLE patients, at least in part through activation of the mTROC1 signaling pathway [15]. To investigate the effect of oxidative stress on EZH2 expression, we treated CD4^+^ T cells isolated from SLE patients with and without H_2_O_2_ (50 μM). H_2_O_2_ significantly increased EZH2 expression in SLE CD4^+^ T cells (Figure 3A). Surprisingly, miR-26a and miR-101 levels were also upregulated by H_2_O_2_ treatment (Figure 3B,C). These results suggest that oxidative stress upregulated EZH2 expression, via mechanisms that might be independent of post-transcriptional regulation by miR-26a and miR-101.

## DISCUSSION

The aim of this study was to explore upstream regulating mechanisms of EZH2 in SLE CD4^+^ T cells. EZH2 is an epigenetic regulator that has been linked to epigenetic changes observed in naïve CD4^+^ T cell as the disease becomes more active in SLE patients [3]. Our model suggests increased EZH2 activity, due to suppressed post-transcriptional regulation, when SLE disease activity is increased [3]. Post-transcriptional regulation of EZH2 is mediated at least in part by miR-26a and miR-101, which are sensitive to glucose availability and are regulated by glycolysis [6]. Further, we have previously demonstrated a pathogenic role for EZH2 overexpression in SLE CD4^+^ T cells. EZH2 mediates abnormal CD4^+^ T cells adhesion in SLE by epigenetic dysregulation of the junctional adhesion molecule A (JAM-A) [4]. Herein, we demonstrate that inhibiting glycolysis or inhibiting mTORC1 signaling attenuates EZH2 expression in CD4^+^ T cells isolated from SLE patients. Our findings also suggest that these effects might be mediated by restoring the expression levels of miR-26a and miR-101, which are downregulated in SLE CD4^+^ T cells compared to healthy normal controls [4]. Post-transcriptional regulation of EZH2 by miR-26a and miR-101 has been well established [16,17]. In addition, we demonstrate that oxidative stress, which stimulates mTORC1 activation, results in EZH2 overexpression in SLE CD4^+^ T cells. Taken together, these data provide a link between activated mTORC1/glycolysis in SLE CD4^+^ T cells and epigenetic dysregulation mediated by EZH2.

It is worth noting that inhibiting glycolysis using 2-DG did not appear to significantly reduce global H3K27me3 levels in SLE CD4^+^ T cells. It is possible that more sensitive assessment tools are needed to detect less robust changes in histone modifications or that H3K27me3 changes induced by 2-DG are site-specific and would be detected using chromatin immunoprecipitation-based methods. Alternative explanations include the possibility that 2-DG might also affect the levels or activity of histone demethylases, compensating for the effect of reduced EZH2 on H3K27me3 levels. Indeed, a crosstalk between H3K27me3 demethylases KDM6A/B and metabolic programs in T cells has been recently reported [18]. It is important to emphasize that EZH2 mediates pathogenic effects in SLE through mechanism that extend beyond its enzymatic function as H3K27 tri-methylase. For example, EZH2 affects DNA methylation and we have previously shown that increased EZH2 leads to DNA demethylation and over expression of JAM-A resulting in pathologic consequences such as increased CD4^+^ T cell adhesion in SLE patients [4]. The effects of specific metabolites in the glycolysis pathway on epigenetic dysregulation in SLE remains to be investigated.

CD4^+^ T cells from SLE patients are characterized by enhanced mTORC1 activity [19], which increases glycolysis through HIF-1α and Myc-related pathways [13]. mTORC1 activation in SLE CD4^+^ T cells promotes Th17 differentiation and suppresses regulatory T cell expansion [19]. This is consistent with EZH2-mediated epigenetic changes in naïve CD4^+^ T cells that were previously observed when SLE becomes more active [3]. Glycolysis is enhanced in T cells from SLE patients and restoring normal glucose metabolism was shown to have therapeutic effects in lupus-prone mice [7]. Further, increased glycolysis was observed in naïve CD4^+^ T cells in lupus-prone mice, and was enhanced following T cell activation [7]. Of interest, recent data suggest that inhibiting glycolysis results in significant reduction of follicular helper T cells (Tfh) in lupus-prone mice [20]. Our previous work in naïve CD4^+^ T cells from SLE patients suggested EZH2-mediated disease activity-dependent progressive epigenetic de-repression in genes encoding BCL-6, ICOS, and CXCR5, which play critical roles in Tfh cell differentiation [3]. Taken together, these data further support the involvement of glycolysis-dependent EZH2 overexpression in the pathogenesis of SLE. These findings also establish a link between increased mTORC1 activation, and thereby enhanced glycolysis, with EZH2-induced epigenetic changes in SLE CD4^+^ T cells.

It has been clearly suggested that oxidative stress is pathogenic in SLE [21]. Depletion of anti-oxidants, such as glutathione, and increased production of reactive oxygen intermediates have been shown in SLE patients [22]. We explored the effect of oxidative stress on EZH2 expression in SLE CD4^+^ T cells for several reasons. First, increased oxidative stress induces mTORC1 activation, and second, almost all environmental triggers associated with disease flares in SLE lead to increased oxidative stress. Furthermore, there is evidence for higher oxidative stress in SLE patients with active compared to inactive disease [23]. Finally, EZH2-mediated epigenetic dysregulation in naïve CD4^+^ T cells from SLE patients that are associated with increased disease activity suggest a role for EZH2 overexpression in lupus flares [3]. We found that oxidative stress, induced by H_2_O_2_ treatment, results in significant upregulation of EZH2. This is consistent with the arguments above. However, H_2_O_2_ treatment also resulted in upregulation of both miR-26a and miR-101. There are three potential mechanisms to explain these paradoxical findings: (1) Oxidative stress can induce EZH2 expression by additional mechanisms independent of our suggested mTORC1/glycolysis/miRNA axis; (2) A negative feedback homeostasis loop may exist to suppress EZH2 via miR-26a and miR-101 as EZH2 levels were significantly increased about 40 folds with oxidative stress; and (3) miR-26a and miR-101 could be directly regulated by oxidative stress independent of mTORC1/glycolysis.

**Figure 4.**
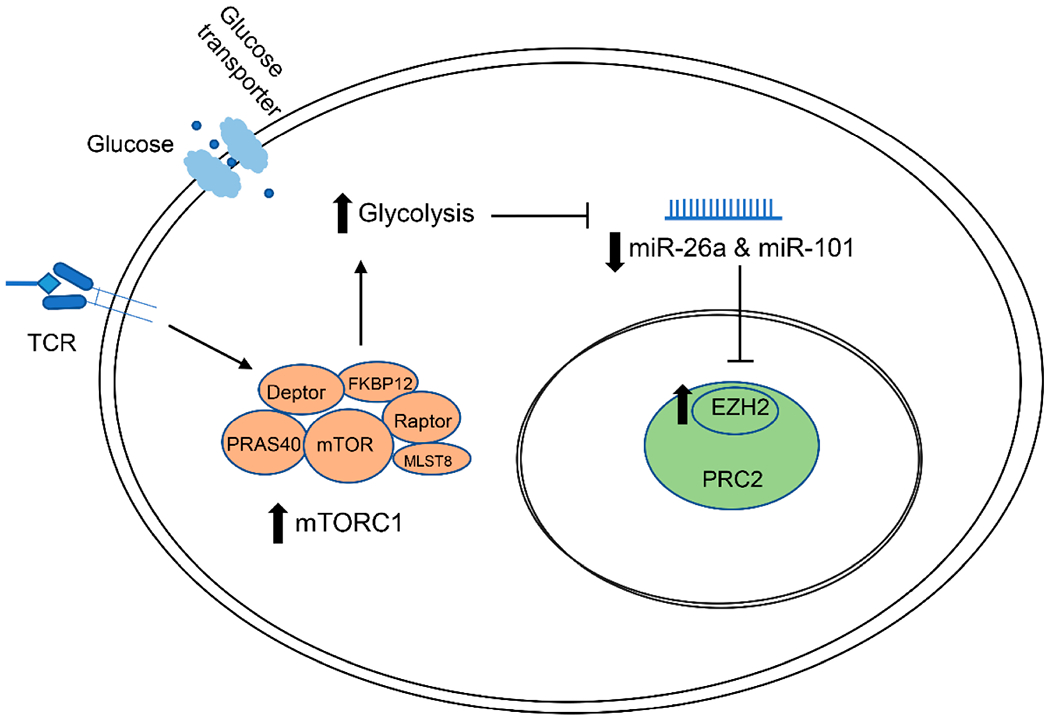
A schematic representation for a proposed pathway showing upstream regulation of EZH2 in SLE CD4^+^ T cells. mTORC1 is activated in SLE CD4^+^ T cells. Activation of mTORC1 promotes metabolic reprogramming and increases glycolysis in CD4^+^ T cells. miR-26 and miR-101 biogenesis is inhibited by higher glycolysis level, limiting post-transcriptional suppression of EZH2 and leading to increased EZH2 levels. TCR: T cell receptor; PRC2: polycomb repressive complex 2.

In summary, our findings suggest that EZH2 overexpression in SLE CD4^+^ T cells is induced by mTORC1 activation and increased glycolysis through effects on post-transcriptional regulation by miR-26a and miR-101 (Figure 4). These data suggest that interaction between immunometabolism and epigenetic regulation might be involved in the pathogenesis and disease flares of SLE. Suppression of EZH2 expression in CD4^+^ T cells might be mediating at least some of the therapeutic effects observed with blocking mTOR activation and with glycolysis inhibitors in SLE and lupus-prone animal models.

## Supplementary Material

Supplementary file
