# Supplementary material for: Increased Expression of EZH2 Is Mediated by Higher Glycolysis and mTORC1 Activation in Lupus CD4+ T Cells
Source: Immunometabolism. Author manuscript; Available in PMC 2020 May 15. (PMC7213603; doi:10.20900/immunometab20200013)
Supplement: Supplementary file [file NIHMS1583625-supplement-Supplementary_file.pdf]

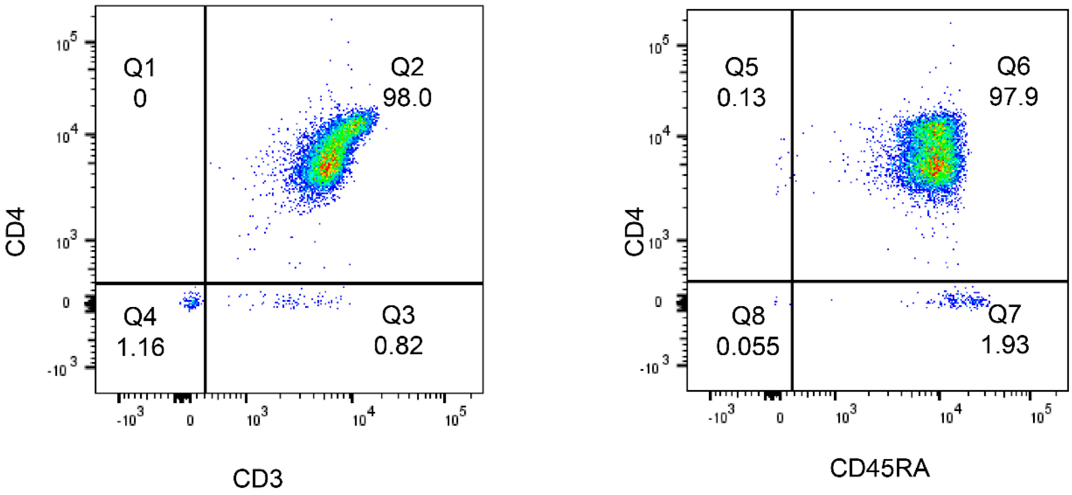

Supplementary Figure S1. A representative flow cytometry analysis demonstrating purity of naïve CD4+ T cells isolated from peripheral blood mononuclear cells from SLE patients used in this study. The purity of naïve CD4+ T cells was assessed by flow cytometry using anti-CD3, anti-CD4, and anti-CD45RA antibodies.

Supplementary Table S1 Demographic information and SLEDAI scores of systemic lupus erythematosus patients recruited in this study.

No.	Age	Sex	Ethnicity	SLEDAI score
1	48	F	European-American	0
2	60	F	European-American	2
3	56	F	European-American	0
4	48	F	European-American	4
5	20	F	Hispanic	0
6	53	F	European-American	8
7	47	F	European-American	2
8	29	F	Asian	12
9	45	F	European-American	0
10	49	F	European-American	0
11	48	F	European-American	6
12	49	F	European-American	0
13	40	F	European-American	6
14	33	F	European-American	6
15	63	F	African-American	2
16	48	F	European-American	5
17	61	F	European-American	2
18	50	F	African-American	2
19	41	F	Native Hawaiian and Other Pacific Islander	0
20	38	F	European-American	4
21	35	F	European-American	6
22	24	F	European-American	0
23	25	F	Asian	6

24	40	F	European-American	6
25	41	F	European-American	2
26	28	F	African-American	10
27	40	F	European-American	0
28	46	F	European-American	2

SLEDAI, Systemic lupus erythematosus disease activity index.
